# Metaheuristic Algorithms Applied to Color Image Segmentation on HSV Space

**DOI:** 10.3390/jimaging8010006

**Published:** 2022-01-05

**Authors:** Donatella Giuliani

**Affiliations:** School of Economics, Management and Statistics, University of Bologna, 40126 Bologna, Italy; donatella.giuliani@unibo.it

**Keywords:** color image segmentation, color image processing, metaheuristic algorithms, firefly algorithm, artificial bee colony algorithm

## Abstract

In this research, we propose an unsupervised method for segmentation and edge extraction of color images on the HSV space. This approach is composed of two different phases in which are applied two metaheuristic algorithms, respectively the Firefly (FA) and the Artificial Bee Colony (ABC) algorithms. In the first phase, we performed a pixel-based segmentation on each color channel, applying the FA algorithm and the Gaussian Mixture Model. The FA algorithm automatically detects the number of clusters, given by histogram maxima of each single-band image. The detected maxima define the initial means for the parameter estimation of the GMM. Applying the Bayes’ rule, the posterior probabilities of the GMM can be used for assigning pixels to clusters. After processing each color channel, we recombined the segmented components in the final multichannel image. A further reduction in the resultant cluster colors is obtained using the inner product as a similarity index. In the second phase, once we have assigned all pixels to the corresponding classes of the HSV space, we carry out the second step with a region-based segmentation applied to the corresponding grayscale image. For this purpose, the bioinspired Artificial Bee Colony algorithm is performed for edge extraction.

## 1. Introduction

Image segmentation is the decomposition of an image into meaningful structures, which is a key step in image processing, with the main purpose of facilitating tasks at higher levels, such as object detection, recognition and classification, in passing from image processing to image analysis and image understanding [1]. The basic goal of any image segmentation process is to subdivide an image into components belonging to different objects or to different parts of an object. Theoretically, pixels derived by the same component should have similar properties, forming a connected region [2]. During recent decades, many color segmentation methods have been proposed in the literature; for an in-depth overview, refer to [3,4,5]. A general and broad classification of segmentation image techniques is reported in [6], where a low-level taxonomy is introduced based on distinguishing segmentation methods into spatially guided and spatially blind, where the former perform the spatial arrangement of pixels, unlike the second ones.

Color segmentation techniques can also be divided into three main categories: feature-space-based methods, image-domain-based methods and physics-based methods [7]. In the first class, cluster segments are generated in such a way that they are homogeneous with respect to the characteristics of the feature space, such as intensity level, color or texture. After mapping pixels into a color space, they are allocated in each cluster based on their features, recurring to predefined similarity criteria. Generally, feature-space techniques are spatially blind; they ignore the spatial distribution of pixel colors. Histogram thresholding techniques can be ascribed to this category. In thresholding techniques, pixels are partitioned according to their intensity or color levels, recurring to a global thresholding or multiple thresholds [8,9]. Histograms are composed of relatively separated parts of intensities, each representing an object of the image. In partitional clustering, segmentation is carried out by partitioning the data into a defined number of clusters; K-means and Fuzzy C-Means algorithms belong to this class [10,11]. Image data can be grouped in a one-dimensional or three-dimensional space, depending on whether it is a grayscale or a color image [12]. In our approach, it is not necessary to predefine the number of clusters, as they will be automatically determined.

Beside homogeneity, a second basic property of segmented regions must be the spatial compactness. Generally, feature-space-based techniques do not take into account the spatial locations of pixels, as they provide a global description of the image. On the contrary, image-domain-based techniques are spatially guided, therefore pixels are clustered based on their spatial relationships, assuming that points of the same object are usually nearby. Region-based, energy-based and edge-based algorithms are included in this class, with the aim to form homogenous and compact groups of pixels from the geometrical point of view. The hierarchical clustering methods are included into this category as well, since they produce image subdivision as nested series of partitions based on a criterion for splitting or merging clusters attempting to group pixels into homogenous regions [13,14]. In fact, superpixel algorithms strongly reduce the juxtaposition between these two categories. Recently, Ren and Malik [15] introduced a novel technique based on superpixels that segments an image into regions by considering their proximity and similarity measures defined using image features. Superpixels replace the rigid pixel structure by delineating regions formed by groups of pixels that look similar, making other processing tasks simpler than using single image pixels.

Principally, color segmentation techniques belonging to the two aforementioned categories are based on monochrome segmentation approaches, operating in a specific three-dimensional color space. In most cases, they are a sort of dimensional extension of grayscale image methods. On the other hand, the methodologies which are part of the third category are diversified according to the physical models applied for describing light interaction with materials. Consequently, they do not correspond to any monochromic segmentation methods. In color image processing, physical models aim at eliminating the effect of highlights and shadowing, [16]. The Healey’s reflection model was one of the first attempts at analyzing the geometrical scene and the physical nature of materials [17].

In the present work, we apply a feature-space-based method in which pixels are identified by the three components of a preselected color space [18]. Consequently, it is very important to define which color space is going to be used because the similarity measure will be defined within it. The quality of segmentation may depend considerably on the employed color space [19]. By using a suitable color space, some segmentation techniques for monochrome images can be extended to segment color images ensuring reliable results. Furthermore, we need to keep in mind that segmentation of color images frequently is viewed as an ill-defined problem, meaning that there are multiple acceptable solutions, due to its intrinsically subjective nature.

This paper is structured as follows: Section 2 is composed of two parts, the introduction of the feature-space-based color segmentation method in Section 2.1 and the implemented region-based method for edge extraction in Section 2.2; Section 3 includes the results achieved applying the two proposed methodologies, respectively, in Section 3.1 and Section 3.2. Finally, we present the conclusion in Section 4.

## 2. Materials and Methods

### 2.1. Color Image Segmentation Method

In this work, a monochromatic-based method was implemented, starting with image decomposition into three different components. After that, each component is processed separately, and finally, the individually achieved results are recombined together [20]. Regarding the selection of color space, we opted for the HSV. In fact, RGB color space is adequate for displaying images but not for image processing, because intensity is not decoupled by chromaticity, hence RGB color space does not produce satisfactory segmentation results [21].

As for the HSV space, we need to clarify that hue is the chromatic feature describing a pure color (red, yellow, etc.), saturation quantifies the amount of gray in a particular color, and generally if saturation appears as a range from 0 to 1, 0 represents gray color whereas 1 is a primary color. Value is the intensity or brightness of the color, where 0 is completely black, and 1 is the brightest. The hue and saturation, or alternatively, intensity, components emulate the human perception of color. More precisely, hue is the dominant wavelength, whereas saturation is its purity, or more specifically, the inverse of the amount of white light contained in the color. The value component is apart from chromaticity, so it is decoupled from hue and saturation.

After defining the color space, an illumination equalization was performed, applying a Gaussian blurring to the value channel of the image with standard deviation sigma equal to 1.5; this gives us a local average for the illumination. We have to keep in mind that color representation is sensitive to illumination, so two colors with the same chromaticity can be recognized as different if they have different lighting intensity. This fact makes the clustering processes inefficient, because pixels from the same class but with different illumination can be identified as pixels from separate classes.

After finishing this preprocessing step, we proceeded to a histogram-based segmentation approach applied to each color channel, which uses a metaheuristic algorithm to automatically define the number of clusters and the histogram maxima [22,23,24,25]. Metaheuristic algorithms are a class of approximate methods that allow us to discover possible solutions by exploring a search space in order to find near-optimal solutions. They are iterative processes developed to search for a solution that is good enough in a time that is small enough [26]. These algorithms are frequently nature-inspired, and they have the advantages of finding global optima due to the action of multiple search agents which are randomly generated [27]. The solution of an optimization problem with a metaheuristic algorithm implies an initialization step generating one or more random solutions. In each iteration step, the current solution is then changed by a new one, created by search operators, with a global optimization approach composed by two schemes: the exploitation of new solutions with the goal of improving the quality of solutions and the exploration of the entire search space to prevent the selection of local optima.

In searching maxima of histogram distributions, we suggest the use of this class of optimization algorithms because they guarantee an enhancement of convergence into global optima despite the presence of numerous local maxima, thanks to the simultaneous action of multiple agents moving randomly all around the research space, [28,29]. To this end, we applied the Firefly Algorithm that employs fireflies as search agents, making use of their idealized flashing characteristics to locate the most significant peaks of grayscale histogram for each component [30,31].

Subsequently, the detected maxima are used as initial values of cluster means for the parameter estimation of a Gaussian Mixture Model [32]. The probability density function of a Gaussian Mixture Model is expressed as a weighted sum of Gaussian density functions, whose parameters are evaluated applying the Expectation-Maximization (EM) technique [33]. The coefficients of the linear combination of Gaussians can be seen as prior probabilities of each component, while the posterior probabilities, derived by the Bayes rule, can be used for assigning pixels to clusters without recurring to a thresholding process. The Gaussian Mixture Model (GMM) is a parametric probability density function represented as a weighted sum of Gaussian component densities. The GMM parameters are estimated from data using the iterative Expectation-Maximization (EM) algorithm. As we know, a univariate Gaussian density distribution is expressed by:

(1) $$p\left(x|\;\mu ,\sigma^{2}\right)=N\left(x|\;\mu ,\;\sigma^{2}\right)=\frac{1}{\sigma \sqrt{2\pi }}exp\left(-\frac{{\left(x-\mu \right)}^{2}}{2\sigma^{2}}\right)$$

where $N\left(x|\;\mu ,\sigma^{2}\right)$ represents the Gaussian or normal distribution of mean μ and standard deviation σ. A Gaussian mixture model is a weighted sum of *K* components of Gaussian densities, analytically given by:

(2) $$p\left(x\right)=\sum_{k=1}^{K}\pi_{k}\cdot N\left(x|\;\mu_{k},\sigma_{k}^{2}\right)$$

Equation (2) represents a linear superposition of Gaussian probability densities and the mixing coefficients $\pi_{k}$ indicates the weight of each distribution. After the initialization phase, the Gaussian parameters $\mu_{k},\sigma_{k}^{2}$ and the coefficients $\pi_{k}$ of the linear combination are evaluated using the Expectation Maximization (EM) algorithm. At each iteration, the EM algorithm computes the responsibilities $\gamma_{k}$, so defined:

(3) $$\gamma_{k}\left(x\right)=p\left(k\left|x\right.\right)=\frac{\pi_{k}\cdot N\left(x|{\;\mu }_{k},\sigma_{k}^{2}\right)}{\sum_{i=1}^{K}\pi_{i}\cdot N\left(x|{\;\mu }_{i},\sigma_{i}^{2}\right)}$$

$\gamma_{k}\left(x\right)$ represents the posterior probability of a given intensity x to belong to the k-th cluster, according to the Bayes rule. In short, the responsibilities estimate the grades of membership indicating the degree to which data points belong to each cluster. Consequently, the assignment of pixels of a given gray level $x_{i}$ to the cluster k is performed by means of the evaluation of the maximum value of responsibilities as k varies, given that, by definition, $\gamma_{k}\left(x_{i}\right)$ indicates the probability of the k-th GMM’s component to have generated the value $x_{i}$.

The issues of the segmentation process applied to each color channel are obtained independently, then they are recombined together to compose the final segmented color image. After that, it performs a reduction in the number of distinct colors recurring to the parallelism of vectors represented in HSV space. In order to do so, we would like to reiterate the fundamental laws of colorimetry, that state [34]:

1.  Any color can be defined by three values and the combination of the three components is unique;
2.  Two colors are equivalent after multiplying or dividing the three components by the same number;
3.  The luminance of a mixture of colors is equal to the sum of the luminance of each color.

According to the second statement, we have chosen the cross product to identify parallel vectors in the HSV space. Indeed, if two vectors have the same direction, or equivalently if they are linearly dependent, their cross product is zero. In this context, if the cross product is approximately null it implies that colors are very similar, so they can be considered as belonging to the same class.

At the end of the process, the performance of a clustering algorithm must be estimated. The academic literature in this field has suggested several performance metrics to assess the validity of cluster partitions [35,36]. Basically, three different techniques are implemented for evaluating the efficiency of clustering algorithms: external criteria, internal criteria and relative criteria [37]. The external validity methods evaluate the clustering results based on their comparison to an externally known result, such as manual image segmentation performed by human users. The internal measures estimate the goodness of a clustering process without considering external information but using the data set itself. Finally, relative clustering validation evaluates the clustering structure by varying different parameter values for the same algorithm (for example changing the number of clusters).

In this work, the algorithm efficiency is computed through an internal clustering validation approach referring to the mean-squared error (MSE) [38]. Usually, MSE is used for assessing distortion between the original image and resulted image. For color images, the formula is extended to include the three components:

(4) $$\mathrm{MSE}=\frac{1}{n\cdot m\cdot p}\sum_{k=1}^{p}\sum_{i=1}^{n}\sum_{j=1}^{m}{\left(I\left(i,j,k\right)-\tilde{I}\left(i,j,k\right)\right)}^{2}$$

where $I\left(i,j,k\right)$, $\tilde{I}\left(i,j,k\right)$ are, respectively, the original and the segmented image, p is the number of the image components (p=3 for color spaces), and n·m is the size of each component. A low MSE value means that the predicted values are close to the real values, practically, the RMSE will be used, defined by: $\mathrm{RMSE}=\sqrt{\mathrm{MSE}}$. This RMSE depends on the orders of magnitude of the observed values. Therefore, it can vary significantly from one application to the next. To resolve this problem, we could consider the relative absolute error (RAE) defined as follows:

(5) $$\mathrm{RAE}=\frac{1}{n\cdot m\cdot p}\sum_{k=1}^{p}\sum_{i=1}^{n}\sum_{j=1}^{m}\left|\frac{I\left(i,j,k\right)-\tilde{I}\left(i,j,k\right)}{\;I\left(i,j,k\right)}\right|$$

Small values of validation indices imply that the estimated image is close to the initial one.

### 2.2. Edge Extraction Applying Artificial Bee Colony Algorithm

After the color segmentation process, we proceed to a region-based segmentation of the corresponding gray image in order to extract edges of homogenous components. The first step in region growing is to select a set of seed points. The region begins to grow from the location of these seeds. In the present work, the selection of initial seed points was achieved through the Artificial Bee Colony algorithm.

The ABC algorithm is a swarm-based metaheuristic algorithm that was introduced by Karaboga in 2005 [39] for optimizing numerical problems. It was inspired by the intelligent foraging behavior of honeybees in nature. The algorithm is specifically based on the model for the foraging behavior of honeybee colonies [40,41]. In the ABC algorithm, the colony of artificial bees consists of three groups: employed bees, onlookers and scouts [25]. Employed bees are those who have discovered a food source. The employed bee whose food source is abandoned becomes a scout bee, starting new random research around the hive. The exchange of information among bees is the most important occurrence in the formation of collective knowledge. Communication among bees related to the quality of food sources takes place in the dancing area of the hive; this dance is called waggle dance. After localizing a source, employed bees share nectar and position information of the food sources with onlooker bees executing the waggle dance. An onlooker bee evaluates nectar information taken from employed bees and decides to employ herself at the most profitable source, with a probability related to the nectar amount [42]. In this context we have adapted the ABC method, considering as food sources the areas of the gray image with pixels not yet assigned to any cluster. Their fruitfulness is greater the greater their extension. The onlooker bees come to the aid of the employed bees in a number proportional to the size of the identified food source and to the number of pixels belonging to it not yet grouped together into any homogeneous region.

## 3. Results

### 3.1. Results of the Color Image Segmentation Method

**Figure 1:**
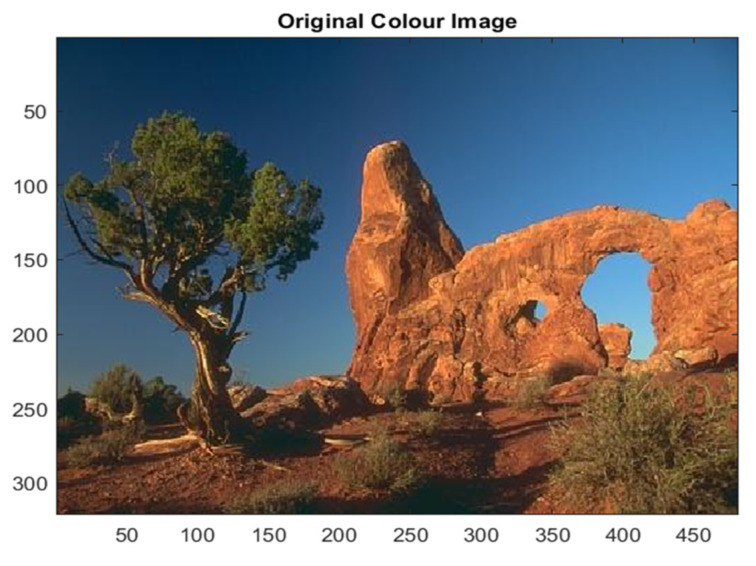
Original image #295087.

As an initial test image, we have considered the BSD image #295087 extracted by the Berkeley Segmentation Dataset BSDS500. The original image contains 61,258 unique colors (Figure 1).

**Figure 2:**
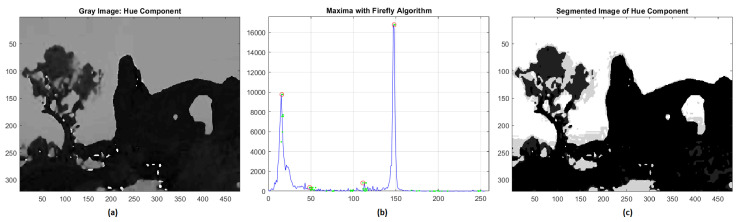
(**a**) Hue component; (**b**) global maxima of histogram distribution by FA; (**c**) segmentation of hue component.

During the segmentation of the hue component, the FA algorithm identified four different clusters of intensities 16, 35, 124, 147, respectively, and the final outcome is shown in Figure 2. As we can see, in the original image two predominant hues appear, the first ranging from ocher to dark brown and the second one is due to the blue sky of the background. The validation of the grayscale segmentation was performed by using the Root-Mean-Square Error (RMSE) and the Normalized Correlation Coefficient (NK) [43,44,45]. For the hue component we have obtained RMSE = 0.0247 and NK = 0.98.

**Figure 3:**
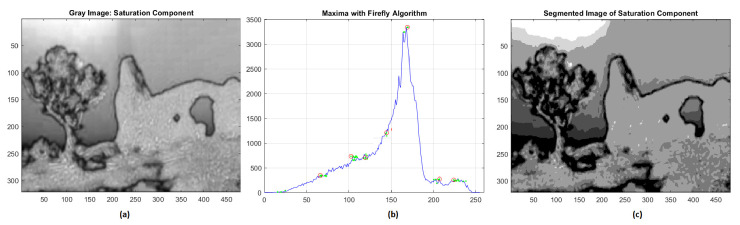
(**a**) Saturation component; (**b**) global maxima of histogram distribution by FA; (**c**) segmentation of saturation component.

The gray distribution of the saturation component is however more complex. The results of the corresponding segmentation are represented in Figure 3, and the evaluated gray levels of the seven clusters are 72, 101, 122, 145, 169, 204, 228 in increasing order. The validation indices are RMSE = 0.0418 and NK = 0.9947, respectively.

**Figure 4:**
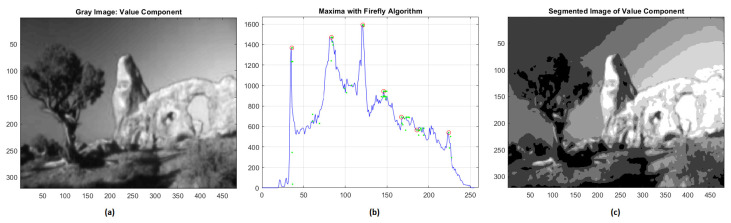
(**a**) Value component; (**b**) global maxima of histogram distribution by FA; (**c**) segmentation of value component.

Regarding the value component, the great variability of the histogram gives rise to seven different clusters with gray intensities equal to 44, 81, 115, 146, 162, 183, 224. Even in this case, we obtained very reliable results with RMSE = 0.0363 and NK = 0.9899 (Figure 4).

**Figure 5:**
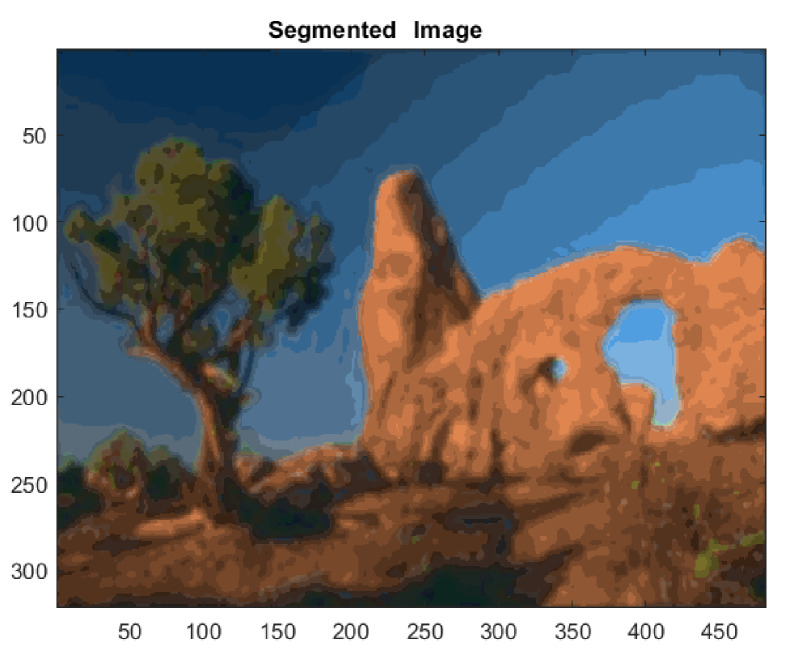
Segmented color image with 132 colors.

After having processed each component separately, we proceed to recombine the images in order to compose the final image, in which 132 different colors are present (Figure 5). The procedure performed a color reduction of 99.7%. For the segmented image, the validation indices, computed with Equations (4) and (5), are RMSE = 0.0618 and RAE = 0.9729, respectively.

**Figure 6:**
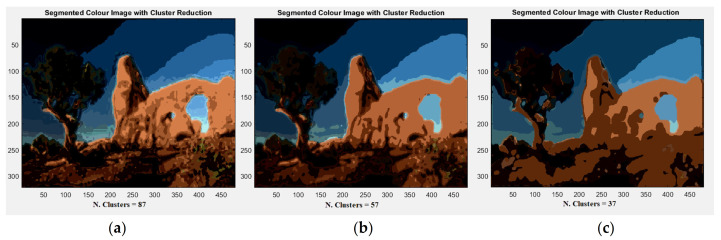
(**a**) Segmented image with 87 colors; (**b**) segmented image with 57 colors; (**c**) segmented image with 37 different colors.

As previously asserted, the variability in adequate solutions for image segmentation is an intrinsic and unavoidable feature, primarily due to the differences in the level of attention, the degree of detail perceived by one human observer compared to another, and the type of represented objects in which the user is interested. However, when pixel colors are projected onto three components, color information are widely scattered, and therefore, one of the drawbacks of color image processing is how to employ this great amount of information. To address this, we performed a color reduction relating to the evaluation of the inner product among vectors in the HSV space. Figure 6 allows us to compare the segmented images after doing cluster reduction. Iteratively applying the procedure, at first we obtained a reduction of 34%, passing from an initial 132 clusters to 87, and then of a further 34.4% with respect to the previous one, reducing the colors to 57 and finally to 37 (Figure 6). Table 1 shows the values of RMSE and RAE from the original image (Figure 1) and the successive segmented ones (Figure 6a–c).

**Figure 7:**
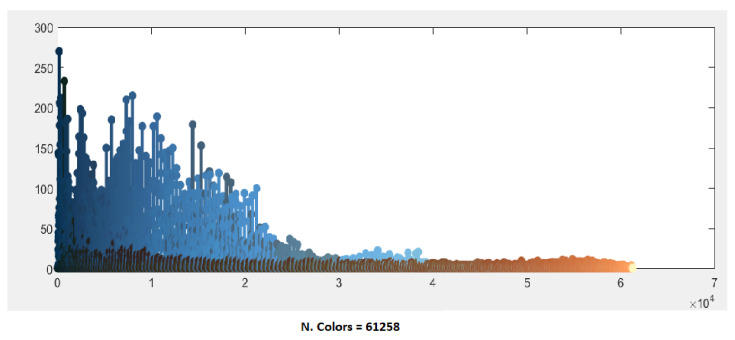
Distribution of the initial 61,258 colors of image #295087.

The BSD image #295087 represents a case with a low color content, essentially blue, brown and green, but with a high texture content, as we can notice by observing the chromatic distribution of the original image shown in Figure 7.

**Figure 8:**
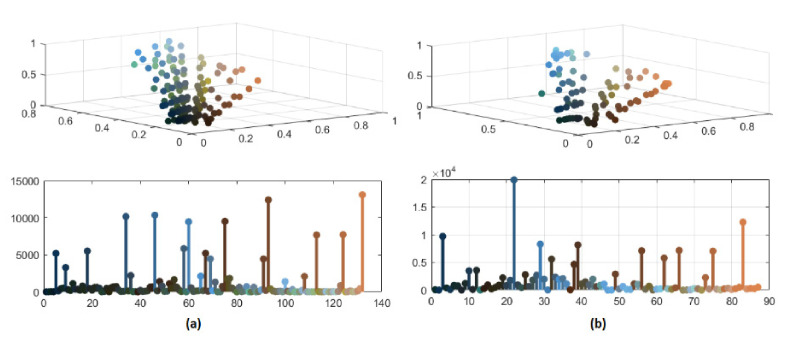
(**a**) Color distribution of segmented image with 132 colors; (**b**) color distribution of segmented image with 87 colors.

**Figure 9:**
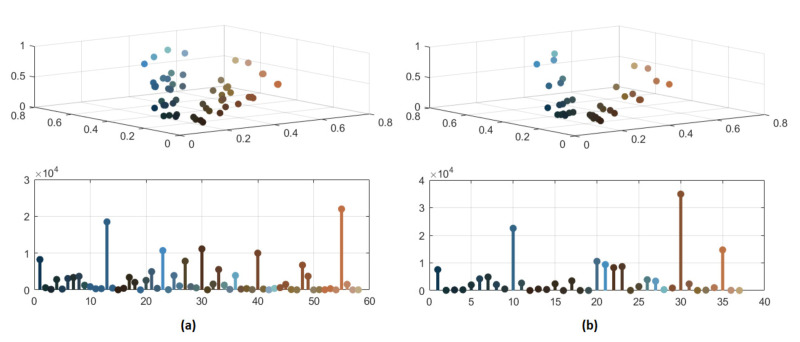
(**a**) Color distribution of segmented image with 57 colors; (**b**) color distribution of segmented image with 37 colors.

In Figure 8 and Figure 9, the significant color reduction is highlighted through the three-dimensional scatter diagrams and the bidimensional chromatic distributions related to the segmented images with 132, 87, 57 and 37 distinct colors, respectively. The relevant decrement of colors may avoid an over-segmentation because of merging pixels with similar colors.

**Figure 10:**
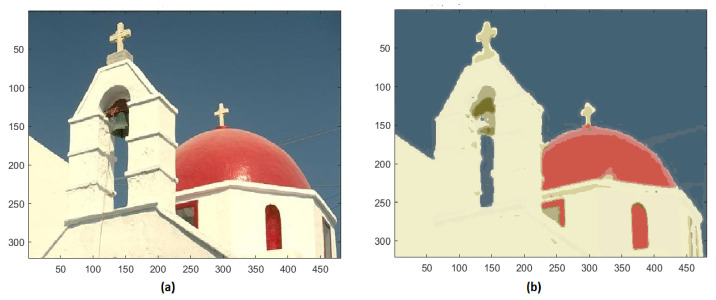
(**a**) Original image #118035 of BSD; (**b**) segmented image with 19 colors.

This method has also been applied to some other images extracted by the Berkeley Segmentation Dataset BSDS500. In the test image #118035 of BSD, the initial 23,786 unique colors are reduced to 19 (Figure 10).

**Figure 11:**
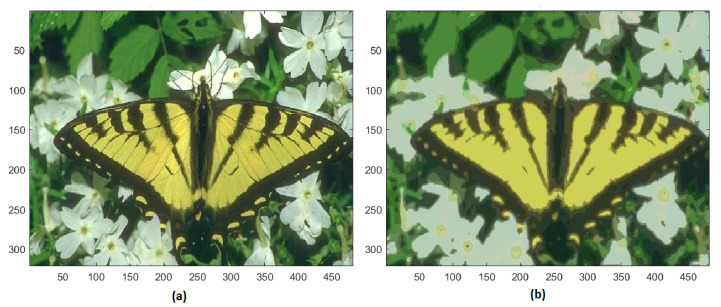
(**a**) Original image #35010 of BSD; (**b**) segmented image with 129 colors.

The training image #35010 of BSD contains 61,267 colors, the final image is represented with only 219 different colors. Nevertheless, the basic chromatic characteristics of the butterfly and the surroundings are preserved (Figure 11).

**Figure 12:**
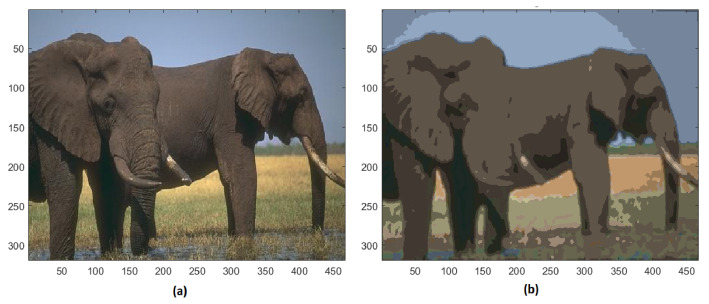
(**a**) Original image #296059 of BSD; (**b**) segmented image with 48 colors.

Analyzing the training image #296059, the initial 27,871 colors are reduced to 48 different colors, and the resulting segmented image is shown in Figure 12. The complexity of the ground texture and the elephant skin is strongly simplified, while the tusks are still clearly distinguishable.

**Figure 13:**
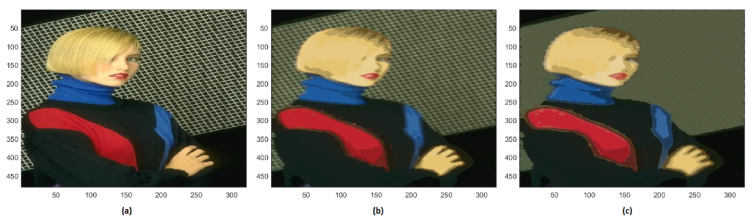
(**a**) Original image #296059 of BSD; (**b**) segmented image with 157 colors; (**c**) segmented image 124 with different colors.

For image #198023 with 31,863 colors, the reduction gives rise to 157 colors. With a further reduction to 124 different colors, the small squares of the grating behind the woman are no longer distinguishable, this makes the foreground more recognizable from the background (Figure 13).

### 3.2. Results of Edge Extraction Applying Artificial Bee Colony Algorithm

**Figure 14:**
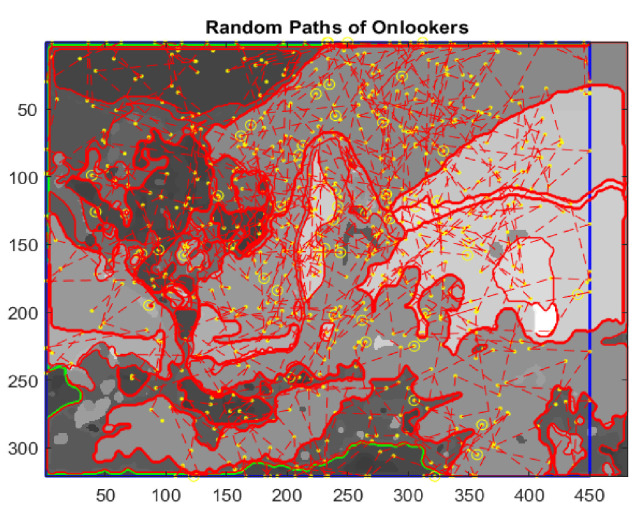
Random paths of onlookers.

In this paper, initially scout bees move in the search space, which is the gray image, describing random paths, each of which is a piecewise linear curve composed by a connected sequence of M arbitrary line segments. The trajectories of scout bees are defined by the following parametric equations:

$$x_{k+1}\left(t\right)=x_{k}\left(t\right)+v_{0}\cdot rand\left(1\right)\cdot cos(rand\left(1\right)\cdot \;\theta$$

$$y_{k+1}\left(t\right)=y_{k}\left(t\right)+v_{0}\cdot rand\left(1\right)\cdot sin(rand\left(1\right)\cdot \;\theta$$

where k=1,…,M, $t\in \left[0;1\right]$, $v_{0}$ is the initial velocity, $\theta \in \left[0;2\pi \right]$ and *rand*(1) is a generator of random numbers uniformly distributed in the interval $\left[0;1\right]$, the end points of each line segment determine the positions of unemployed bees during their flights. Initially, the paths will be confined inside the image space, so as not to go beyond edges. If along the path a scout bee finds a food source, which is a zone with unclassified pixels, the growing process will be activated starting from the actual position, otherwise the bee keeps going undisturbed. Once the region with uniform gray intensity is outlined, its edges are extracted and the bounded box of boundaries is determined (Figure 14).

**Figure 15:**
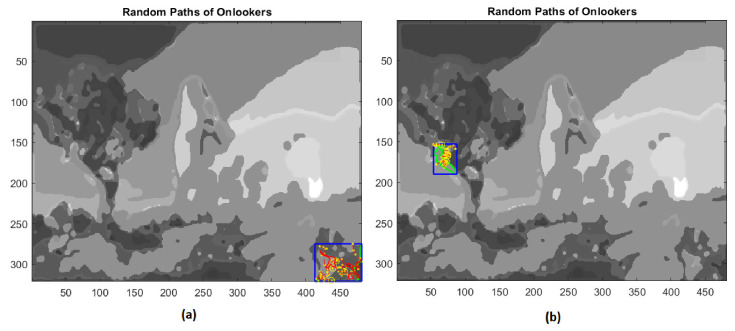
(**a**) First example of local search by onlookers in a rectangular area (blue line); (**b**) second example of local search by onlookers on test image.

**Figure 16:**
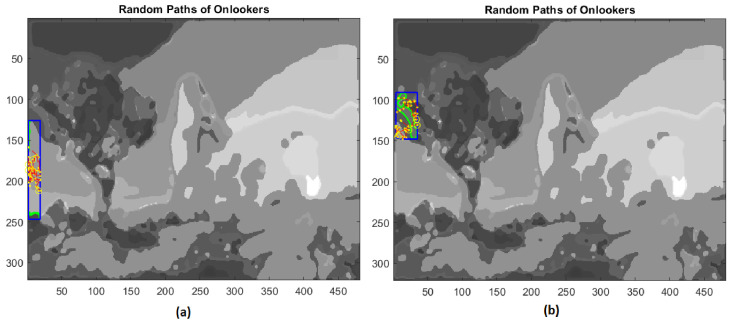
(**a**) Third example of local search by onlookers in a rectangular area (blue line); (**b**) forth example of local search by onlookers on test image.

At this point, employed bees share their food source information with onlooker bees waiting in the hive and then onlooker bees choose their food sources depending on this information. The scout bees come back to the hive for executing the waggle dance in order to involve onlooker bees in the exploitation phase. In the present application of the ABC algorithm, the fitness values are computed through the percentage of pixels not yet assigned and included inside the bounded box of the extracted regions. Then, onlooker bees give rise to a local search, rushing to scouts’ aid proportionally to the number of unclassified pixels and to the size of the rectangle containing the extracted boundary (Figure 15 and Figure 16).

**Figure 17:**
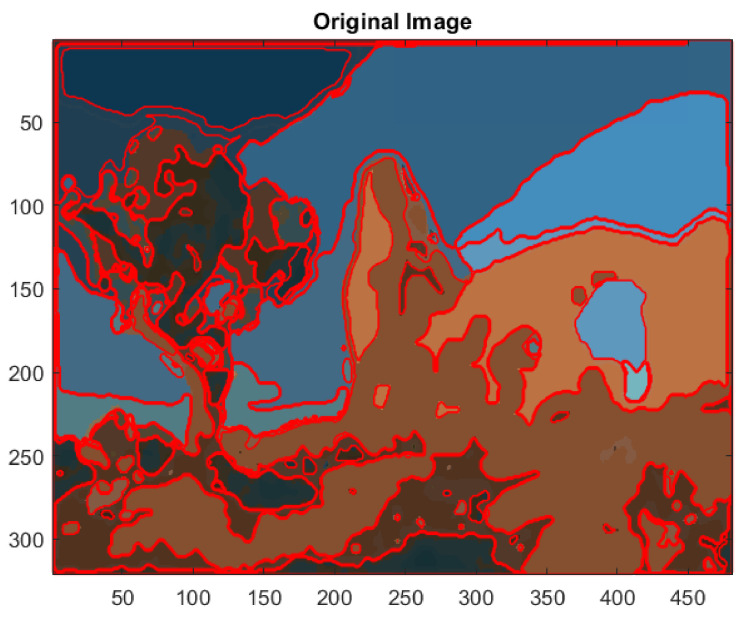
Edge extraction of the segmented image #295087 of BSD with 37 clusters.

The extracted edges of the segmented image #295087 of BSD with 37 clusters are displayed in Figure 17. The algorithms, developed with Matlab, are able to detect even low significance regions that eventually could be excluded on the basis of the measure of their area or perimeter length.

## 4. Discussion

This work performed a color image segmentation, referring to metaheuristic and nature-inspired algorithms. The algorithms are applied to hue, saturation and value components separately. Thus, this method pertains to the category of monochrome segmentation approaches, which can be considered as a dimensional extension of grayscale image methods.

The metaheuristic Firefly Algorithm automatically evaluates the number of clusters and their initial centroids. Subsequently, the outcomes of FA are used as initial means to estimate the Gaussian Mixture Model. The multilevel image is obtained by recombining the three segmented components. A further color reduction is performed through the use of the inner product, as an index of similarity among colors. The validation analysis has been carried out using different standard measures, showing that the method is fairly robust and reliable.

Concerning the spatial segmentation, the application of the probabilistic ABC algorithm carries out boundaries of segmented regions in a fast way. The erratic motion of scout bees makes it possible to detect edges even if the size of the regions is very small. This is due to the local search activated by onlookers rushing to scouts’ aid that makes the research more effective and detailed. In this context, the region-based approach is performed on the segmented grayscale image rather than on the color one. This is due to a limitation of the method hereby applied, which will have to be overcome in future research. While Balasubramanian et al. [46] applied the region-growing method on color images with a dynamic color gradient thresholding, in this research, the choice of operating with grayscale images is crucial for the application of the ABC metaheuristic algorithm in image processing, which is also one of the aims of the present work.

## Figures and Tables

**Table 1:** Root-mean-squared errors and relative absolute errors.

Number of Clusters	RSME	RAE
132	0.0618	0.9729
87	0.0690	1.0087
57	37.0155	0.9960
37	37.2132	0.9963

## Data Availability

The images analysed in this research were taken from the Berkeley Segmentation Dataset BSDS500. Available at: https://www2.eecs.berkeley.edu/Research/Projects/CS/vsion/bsds/BSDS300/html/dataset/images.html, accessed on 10 December 2021.
